# Ecstatic Epileptic Seizures: A Glimpse into the Multiple Roles of the Insula

**DOI:** 10.3389/fnbeh.2016.00021

**Published:** 2016-02-17

**Authors:** Markus Gschwind, Fabienne Picard

**Affiliations:** ^1^Department of Neurology, University Hospital and Medical School of GenevaGeneva, Switzerland; ^2^Functional Brain Mapping Laboratory, Department of Neuroscience, Biotech Campus, University of GenevaGeneva, Switzerland

**Keywords:** ecstatic, epilepsy, bliss, self-awareness, insula, time dilation, predictive coding, salience

## Abstract

Ecstatic epileptic seizures are a rare but compelling epileptic entity. During the first seconds of these seizures, ecstatic auras provoke feelings of well-being, intense serenity, bliss, and “enhanced self-awareness.” They are associated with the impression of time dilation, and can be described as a mystic experience by some patients. The functional neuroanatomy of ecstatic seizures is still debated. During recent years several patients presenting with ecstatic auras have been reported by others and us (in total *n* = 52); a few of them in the setting of presurgical evaluation including electrical brain stimulation. According to the recently recognized functions of the insula, and the results of nuclear brain imaging and electrical stimulation, the ecstatic symptoms in these patients seem to localize to a functional network centered around the anterior insular cortex, where we thus propose to locate this rare ictal phenomenon. Here we summarize the role of the multiple sensory, autonomic, affective, and cognitive functions of the insular cortex, which are integrated into the creation of self-awareness, and we suggest how this system may become dysfunctional on several levels during ecstatic aura.

## Introduction

**Figure 1:**
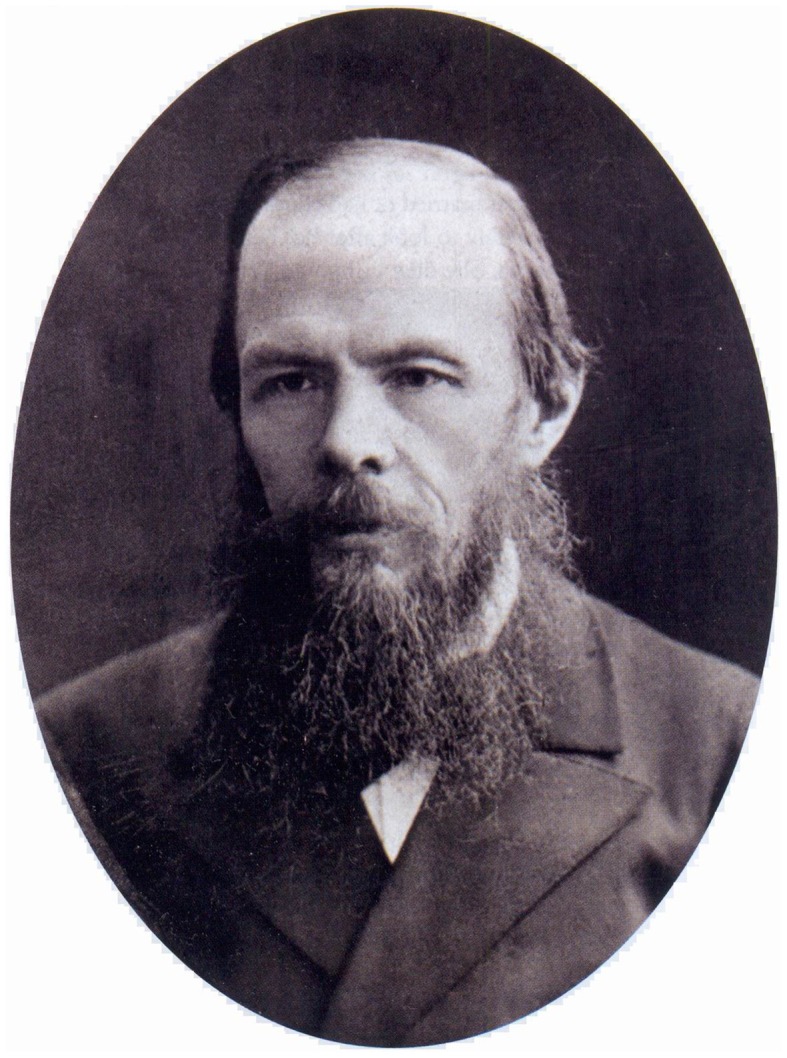
**Fyodor Dostoevsky, 1871^1^**.

Ecstatic epileptic seizures are described as an overwhelmingly positive experience. Patients have trouble finding appropriate words (Cirignotta et al., 1980), and sometimes give simplified descriptions (e.g., “like bubbles rising in the head,” “feeling of warmth in the whole body”). The interested epileptologist, in order not to miss these rare cases, needs to actively ask for key symptoms in the patient. The patient's descriptions of ictal episodes with strong emotional disturbances depend to a large extent on the patient's power of introspection, intelligence and vocabulary (Williams, 1956). The fearful apprehension of the imminent complex focal or secondary generalized tonic-clonic seizure can intermingle with the bliss of the ecstatic aura after the first seizure. Such personal feelings, as the “hallucination of emotion” (Williams, 1956), can seem so abnormal, that patients are often reluctant to communicate them. Overall, frequency of ecstatic seizures is therefore probably underestimated and historical documentation remains scarce (Picard and Craig, 2009). Russian novelist Fyodor Dostoevsky's testimony (Dostoevsky, 1869, 1872) (Figure 1) can be considered the first description of ecstatic auras in literature (Picard and Craig, 2009).

Moreover, the existence of ecstatic seizures was initially even denied by some leading epileptologists (Penfield and Kristiansen, 1951; Gastaut, 1978; Hughes, 2005), for review see also (Baumann et al., 2005; Rossetti, 2006).

Ecstatic seizures belong to the focal epilepsies, of which origin is limited to regions of one hemisphere. Consciousness is preserved during the ecstatic aura, the first, subjective part of the seizure, which is often imperceptible by witnesses. Epileptic seizures are, indeed, dynamic phenomena, and as in other focal seizures, after the aura, the seizure can evolve into impairment of consciousness, sometimes with oral or gestural involuntary automatisms, and in the extreme, into a generalized tonic-clonic seizure.

We defined ecstatic seizures as seizures fulfilling the following criteria: the feeling of (1) intense positive emotion (bliss), (2) enhanced physical well-being, (3) heightened self-awareness or heightened perception of the external world (clarity). BOX 1 provides an exemplary synthesis of our patients' testimony during the last 10 years [cited from Picard and Craig (2009), Picard (2013), and Picard and Kurth (2014)]. These symptoms should occur as primary experiences and should not be caused by other ictal symptoms, e.g., pleasant complex hallucinations.

Box 1 **Our patients' testimony**

***1. Intense serenity and bliss.***

– “*This led to a feeling of complete serenity, total peace, no worries; it felt beautiful, everything was great.”*
– “*The immense joy that fills me is above physical sensations.”*
– “*It is a feeling of total presence, an absolute integration of myself, a feeling of unbelievable harmony of my whole body and myself with life, with the world, with the ‘All’.”*

***2. Enhanced physical well-being.***

– “*It was something that I have never felt before. It felt as though my body was filling up with a sensation which was quite surreal. The feeling was almost out of this world.”*
– –*“[…] a halo, something pleasant which fills my inner body, wrapping me, with a rapid crescendo. It is a well-being inside, a sensation of velvet, as if I were sheltered from anything negative. I feel light inside, but far from being empty. I feel really present. Something has taken possession of my body, to feel really good…”*

***3. Heightened self-awareness and/or perception of external world.***

– “*During the seizure it is as if I were very, very conscious, more aware, and the sensations, everything, seems bigger, overwhelming me.”*
– “*I feel rooted to the spot with a more developed consciousness. I feel a stronger consciousness of the body and the mind, but I do not forget what is around me.”*
– “*It affects both the cerebral thought, which is very intense and concentrated on itself, and the physique.”*
– “*Being very conscious of myself, I feel discharged from anything else, although I do not lose consciousness.”*
– “*I feel very, very, very present at that time; the consciousness of myself is very increased, rather on a psychic point of view. I am one hundred percent concentrated on myself.”*

***4. Feeling of dilated time.***

– “*I escape into the time space of my body. It is a moment of fullness in the loophole of time, a return to myself.”*
– “*Entirely wrapped up in the bliss, I am in a radiant sphere without any notion of time or space. My relatives tell me that it lasts two to three minutes, but for me these moments are without beginning and without end.”*

***5. Feeling of overload.***

– “*It is a physical state, an overload. The feeling is intense, with a sensation of fullness.”*
– “*The sensation is certainly more intense than could be achieved with any drug.”*
– “*This feeling became stronger and stronger, until it became so strong that it was unbearable and led to a loss of consciousness.”*

***6. Mystic/religious experience.***

– “*Maybe the closest sensation that I know would be an orgasm, but what I felt was not at all sexual. I have no religious feeling, but it was almost religious.”*
– “*These experiences brought me confidence. They confirm that there is something that surpasses us.”*
– “*It is a big happening in your life to have these seizures. Thanks to these experiences, I do not fear death anymore. I see the world differently.”*

***7. Anxiety.***

– “*…soon after the very first seizures, an anxiety intermingled very rapidly with the bliss sensation”*
– *A patient described anxiety because of the anticipated fear of how he would appear to other people during his complex focal seizures. However, as the bliss increased, it overcame the associated anxiety.*

Recently, it has been proposed that ecstatic epileptic symptoms may localize the beginning of the seizure (or at least the “symptomatogenic zone,” which can be a zone of propagation from the true seizure onset zone) in the anterior-dorsal insular cortex (Picard and Craig, 2009). This interpretation appears consistent with recent findings on the functions of this brain region, which we will present in the following sections.

## Epileptologic approach of ecstatic seizures

### Patients in the literature

Up to now, a total of 52 patients experiencing ecstatic auras have been described in the literature (Alajouanine, 1951; Penfield and Kristiansen, 1951; Mulder and Daly, 1952; Subirana and Oller-Daurella, 1953; Feindel and Penfield, 1954; Williams, 1956; Mullan and Penfield, 1959; Boudouresques et al., 1972; Cirignotta et al., 1980; Naito and Matsui, 1988; Morgan, 1990; Cabrera-Valdivia et al., 1996; Vuilleumier et al., 1997; Vera et al., 2000; Asheim Hansen and Brodtkorb, 2003; Isnard et al., 2004; Stefan et al., 2004; Landtblom, 2006; Picard and Craig, 2009; Landtblom et al., 2011; Picard, 2013; Picard et al., 2013b; Surbeck et al., 2013; Ronchi et al., 2015). Table 1 summarizes each of these patients together with the essential ecstatic semiology, associated symptoms, results from brain imaging and electroencephalogram (EEG) recording and, when known, the etiology.

**Table 1:** **Description of the cases of ecstatic seizures reported in the literature**.

**Report**	**Age, sex**	**Ecstatic semiology**	**Associated symptoms**	**Localizatory aspects**	**Remarks**	**Left/right**
Penfield and Kristiansen, 1951	1 7 years, m (case H.M)	1. “Sensation of joy” in the epigastrium	2. Perceptiual illusion	EEG: epileptiform left mid-temporal	Symptoms started after (sic) epilepsy surgery in two left frontal gyri for partial seizures	Left
Alajouanine, 1951	25 years, f	2. Suave feeling rises. 3. Wings take her to the sky, feels happiness and liberty	1. Epigastric aura, 4. sweet and pleasant shudder in left body half	EEG: bilateral temporal spike and waves	Neurosyphilis	Rather left
Mulder and Daly, 1952	23 years, f	1. Pleasant butterfly in stomach. 5. Euphoric and talkative	2. Right head deviation 3. got blank. 4. chewing automatisms, 6. purposeless movements with both hands	X-ray: calcified lesion in deep temporal region	Tumoral lesion	Right
Subirana, 1953	44 years, m	1. Sudden feeling of happiness “complete out of this world”	–	EEG: focal temporal slowing	Glioblastoma–removed	Left
	45 years, m	1. Indescribable happiness “knew what it was like to be in heaven” - impression that it lasts for hours	People appeared completely different	EEG: left anterior temporal slow waves and spikes		Left
Feindel and Penfield, 1954	(case 6)^*^)	“Slightly like going into a trance.”—"Time and space seem occupied	Confuse sensation in head	Stimulation: right medio-temporal /insular border		Right
Williams, 1956	41 years, f (case 35)	Sudden feeling of being lifted up, elation, satisfaction, most pleasant sense, ≪ knowledge no one else shares ≫, between life and dead	Palpitations, pale and trembling,	?		?
	32 years, m (case 36)	Sudden feeling of extreme well-being in all senses. …“as if in another world”	Pleasant epigastric sensation, beautiful colors and indescribable taste	Cortical atrophy		Left
Mullan and Penfield, 1959	25 years, m (case 38)^*^)	2. Illusion of greater awareness, “new awareness” (of smells, sounds, visible objects, and pressures)	1. Rising epigastric aura; 3. unconsciousness; 4. mastication; 5. automatisms	Electrical Stimulation: right anterior insula, 1 cm. deep. produced a sensation in his right foot. (felt, as he did in his attacks, an increased awareness)		Right
Boudouresques et al., 1972	20 years, m	2. Like being high on drugs, 4. ineffable beatitude, calm euphoria, bliss,	1. Epigastric anxiety, 3. déjà-vécu, 5. urge to defecation – a gesture which was not foreseen in the seizure program could suspend the fit	Gaseous encephalography: bilateral hippocampal calcifications	Urbach-Wiethe Syndrome	
Cirignotta et al., 1980	30 years, m	Ineffable joy. Intense pleasure without match in reality (perhaps music). Absence of all disagreeable feelings	Psychomotor arrest	EEG: Right temporal spikes during ecstatic seizure		Right
Naito and Matsui, 1988	6 2 years, f	Extreme happiness as if she was in paradise	Tears of happiness. Sees halo around god	EEG. Left anterior spikes during sleep		Left
Morgan, 1990	38 years, m	3. feels at the source of knowledge and understanding, 4. time dilation	1. prompt irritation and detachment 2. bright dazzling light and sees Jesus Christ	CT/surgery: Lesion in inferior lateral temporal lobe	Astrocytoma	Right
Cabrera-Valdivia et al., 1996	25, f	Internal peace, calmness, intense (non-sexual) pleasure, like being drugged	Rares generalizations	MRI normal EEG during television proximity: generalized spikes and polyspike-wave complexes	Episodes induced by close proximity to television (self-induction)	
Vuilleumier et al., 1997	37 years, f	Feeling of consciousness of everything. Heautoscopy - astral travel, intense ecstasy	Brief epigastric sensations	MRI normal. EEG: generalized parietal spike and waves and 4Hz temporoparietal spike-and-wave (both right>left)		Right
Vera et al., 2000	45 years, m	2. Feeling union with the whole world and with god	1. Sees flickering blue lights	No EEG	right occipital AVM (=>removal stopped ecstasy seizures) Hippocampal sclerosis was removed years later	Right
Ostrowsky et al., 2000	?, ?	Pleasant experiential phenomenon reported as a weird feeling of “flying away”	?	?	Electrical stimulation of insular cortex	?
Asheim Hansen and Brodtkorb, 2003	23 years, m (case 1)	Indescribable trance of pleasure	Cold shivers, muscle tension, delicious taste. automatic swallowing – post-ictal aphasia	MRI: normal EEG: normal		?
	55 years, m (case 2)	3. Profound relaxation	1. Jerks in right face; - 2. grin – often occurred when passing a particular place. - recalling former fits could trigger fits	MRI: Left parieto-occipital atrophy EEG: epileptiform left temporal		Left
	62 years, f (case 3)	1. Overwhelming warm, wooly, pleasant feeling spreading through the skin - ^†^)	2. Post-ictal headache and nausea	None		?
	41 years, f (case 4)	1. Intense sensation in stomach (“helplessly in love”). 2. voice comes from god (while agnostic)	2. Sometimes heard voices 3. anxiety and fear accompanied by shuddering and an urge to swallow	MRI: normal EEG. unspecific fronto-temporal (right ?)		Right?
	37 years, f (case 5)	Feeling of unfamiliarity with her surroundings accompanied by a vague erotic component. delightful wooly feeling in her head. ^†^)	Buzzing sound in ears, Ictus emeticus (before surgery) – disappeared after surgery	EEG: epileptiform right frontotemporal	Hippocampal sclerosis	Right
	51 years, m (case 6)	Clairvoyance feeling of a “telepathic contact with a divine power”	Like “twinkling polar light” in his pelvic region	Normal MRI EEG: epileptiform left ? frontotemporal		Left
	41 years, f (case 7)	2. Intense happy feeling,	1. crossing a line as if the world was divided; smell of sawdust and a stereotyped image of herself as a child superimposed on her real vision 3. Sees her grandfather during fit	None		?
	53 years, f (case 8)	2. Delightful sensation of “inebriation and floating” and she feels that her mind leaves the body	1. Thirst and urge to swallow; hears music.—post-ictal dysphasia and urge to urinate	MRI normal EEG: epileptiform left frontotemporal		Left
	30 years, f (case 9)	sees wise woman who presents to her ultimate mission of life. Unable to interpret the details, but seems extremely important. - She admits dose reduction of her AED to enhance seizures	Strange music, harsh taste, numb prickling sensation in her right arm	EEG: Left temporal seizure onset	left mesial temporal sclerosis	Left
	48 years, f (case 10)	Sudden indescribably pleasant and joyous feeling	Faintness and oral automatisms	MRI: left temporal lesion (surgery) EEG: Epileptiform right temporal	Low grade glioma removed from left temporal lobe, but seizures persisted. right HS	?
	48 years, m (case 11)	Erotic aspect, starts in stomach and spreads upwards. “Like an explosion.” - Peculiar unification to others	Senses red and orange colors without seeing the color, déjà-vu, queer taste and gooseflesh	MRI: Lesion in right temporal lobe	anaplastic oligodendroglioma	Right
Isnard et al., 2004	23 years, m^*^	Mirth, clairvoyance	Orofacial and bodily somatosensory symptoms, throat and dysarthria, preserved contact	MRI right parietal cortical dysplasia and schizencephaly SEEG: posterior insula with propagation in temporal operculum and parietal operculum	cortical dysplasia and schizencephaly	Right
Stefan et al., 2004	26 years, m (case 1)	1. Pleasant feeling in head; intimacy feeling and feeling of happiness	2. Staring gaze (interictal depression)	MRI: Right tumor hippocampus and parahippocampus	Astrocytoma	Right
	56 years, m (case 2)	2. Hot pleasant feeling in head arising. Feeling like orgasm	1. Unpleasant smell 3. staring gaze, swallowing	MRI: Right hippocampal atrophy	HS	Right
	29 years, f (case 3)	1. Strange feeling (lightly dozy) of pleasant security “like orgasm”	2. Seeing of letters and signs; 3. after that, fear 4. Loss of consciousness, oral automatisms	MRI: gyrus occipitotemporal lesion	Oligodendroglio- ma of left occipi- totemporal gyrus	Left
	36 years, m (case 4)	1. Strange, pleasant, rising feeling, mainly by coldness or fever	2. Clouding of consciousness with staring gaze, chewing movement (interictal depression)	MRI: left hippocampal atrophy	HS	Left
	36 years, m (case 5)	1. By eating of sharp spices and sauces; orgasm-like feeling with prickling in the perineum	2. Loss of consciousness with staring gaze; right arm is rubbing on left arm; ictal speech	MRI: right hippocampal atrophy	HS	Right
	43 years, f (case 6)	1. Pleasant feeling, euphoria, safe feeling, feeling of flying	2. Fear, cold shiver, depression 3. staring gaze, oral automatisms	MRI: parietal atrophy	Parietal atrophy, bilateral with right predominance	Right
	56 years, f (case 7)	1. From chest; rising pleasant feeling in head; followed by (ictal) depression	2. Staring gaze and pain 3. Automatisms and postictal aphasia	MRI: left temporomesial. lesion	Cavernoma	Left
	60 years, m (case 8)	1. From stomach; in head, rising pleasant feeling	2. Visual field is changing; contortion of faces 3. Déìjà-vu experiences, staring gaze, oral automatism	MRI: right parahippocampal lesion	Ganglioglioma, parahippocampal + mesial right	Right
	58 years, f (case 9)	Dizzy and hot feeling in head with pleasant feeling, happy	(Interictal depression)	MRI: Temporal (HS ?)	Gliosis	Right
	42 years, m (case 10)	Pleasant feeling, and feels euphoria	1. Hearing slightly 2. staring gaze, oral automatism. (interictal depression)	MRI: Temporal tumor	Astrocytoma	Left
	36 years, m (case 11)	Pleasant feeling	Epigastric	MRI: Temporal lateral-basal lesion	Right temporal DNET	Right
Picard and Craig, 2009	53 years, f (case 1)	Never-felt surreal warmth, filling up her body from her feet to her head, complete serenity, almost religious		MRI: Left temporopolar lesion with compression of surroundings	Meningioma	Left
	37, m (case 2)	“Unalterable bliss, escape into the time space of my body.” “Moment of fullness in the loophole of time.”	Déjà vu	MRI/surgery: Right parahippocampal lesion Ictal EEG: 1. right anterior temporal rhythmic elements (7-8 Hz), 2.diffuse flattening, 3. diffuse slow waves, 4. right temporal delta slow waves	Tumoral mass	Right
	25, m (case 3)	Sensation of intense well-being, becoming stronger and stronger, until being unbearable, leading to loss of consciousness	Sensation of loss of balance with gaze fixation difficulties	Normal MRI. Interictal EEG: rare theta slow waves in the left anterior temporal area		Left ?
	36, f (case 4)	Intense pleasant feeling, heightened self-consciousness, feels discharged from anything else. Concomitant warmth rising in her body up to her head,	Tachycardia	Normal MRI. Interictal EEG: bursts of left anterior and midtemporal sharp theta slow waves		Left
	64, f (case 5)	Well-being of almost spiritual consonance	Post-ictal jargonophasia - A joy or relief can trigger seizures	MRI: Lesion in left temporal pole region. interictal EEG: left anterior temporofrontal epileptiform activity	Meningioma	Left
Landtblom, 2006; Landtblom et al., 2011	35, m	1. Pleasant feeling that someone stands behind him, with a distinct wish to support and comfort, wherever he goes	2. State of altered consciousness, nausea, irritation of the throat, and urge to urinate	MRI: left hippocampal atrophy, ventricular asymmetry. EEG: seizure occurs at most medial subtemporal electrode on the left side SPECT: left anterior insula activation		Left
Carrazana and Cheng, 2011	77, m	1. Aura of bright, “beautiful,” and expanding light appears over his left side. Sense of being “calm,” “at peace,” like a tunnel, into which his soul is transported. Unconditional love, God	2. Impaired consciousness for several seconds, 3. violent head and body turn toward the left side, and generalized clonic movements	MRI: large encephalomalacia involving the right temporal lobe with ex-vacuo dilation of the right lateral ventricle. EEG: focal slowing, poorly defined sharp waves over the right frontotemporal area	Car accident causing right temporal depressed skull fracture and bilateral subdural hematomas	right
Picard, 2013	17 years, m (case 1)	Sudden understanding and meaningfulness, time dilatation	Gustatory hallucinations. seizures were all triggered by a pleasant context	MRI: lateral temporal pole tumoral lesion PET hypometabolism in the temporal pole, mesial and anterior lateral temporal lobe and anterior insula on the right side. SPECT (SISSCOM) ictal hyperperfusion at the junction of the right dorsal mid-insula and central operculum, and in the right anterior temporal region	Ganglioglioma in lateral temporal pole	Right
	39 years, m (case 2)	Like a continuous series of profound “aha!” moments.” Everything is joined together into one whole, certainty immune to rational doubt		MRI normal		?
Picard et al., 2013b	23 years, f^*^)	Intense feelings of bliss and well-being – enhanced sensory perception with intense perception of colors – subjective time dilation	Sensation of airflow that left her stomach with a feeling of “floating” – post-ictal loss of consciousness with gestural and oroalimentary automatisms	MRI normal. EEG: spontaneous seizure in right mesiotemporal cortex	=> Electrical stimulation of right anterior insula causes symptoms	Right
Surbeck et al., 2013	49 years, f^*^)	Orgasmic feeling^a^)	Visual symptoms (flashing lights), déjà vu	MRI: discrete atrophy of the left hippocampus EEG: bilateral temporal spikes	Electrical stimulation triggered the symptoms => left hippocampus at 3 mA. with 18-s afterdischarge over the left hippocampus, parahippocampal gyrus, and anterior-inferior insula. => right hippocampus at 1 mA with a 45-s seizure discharge over the right hippocampus, parahippocampal gyrus, temporal pole, and anterior insula	?
Ronchi et al., 2015	43 years, m	short euphoric states	Generalized tonic-clonic seizure	MRI: neoplastic lesion, affecting the entire right insula	Neoplastic	Right

The numbers in columns “ecstatic semiology” and “associated symptoms” represent the chronological order of the symptoms during seizure.

a According to oral communication with the authors this patient described only the “mental part” of an orgasmic feeling, however, this was not specified in the report.

† These patients probably presented only the physical aspect of well-being.

* In these patients electrical stimulation could reproduce an ecstatic phenomenon.

SEEG, stereo-electroencephalographic electrode implantation; HS, Hippocampal sclerosis; DNET, dysembryoplastic neuroepithelial tumor; SPECT, Single-photon emission computed tomography; SISCOM, Subtracted ictal SPECT, coregistered with MRI (O'brien et al., 1998).

Among the patients reported in Table 1, we note several case reports in the 1950s, the time when EEG and brain surgery started to form a more specialized field of epileptology (Alajouanine, 1951; Penfield and Kristiansen, 1951; Mulder and Daly, 1952; Subirana and Oller-Daurella, 1953; Feindel and Penfield, 1954; Williams, 1956; Mullan and Penfield, 1959). The topic regained interest in the late 1990s, with the advent of MRI (Vuilleumier et al., 1997; Vera et al., 2000; Asheim Hansen and Brodtkorb, 2003; Isnard et al., 2004; Stefan et al., 2004). However, only in recent years, have technical advances of multimodal brain imaging provided new possibilities for localization of the epileptogenic region using MRI (Wagner et al., 2011; Hong et al., 2014), electrical source imaging (Brodbeck et al., 2011; Megevand et al., 2014), combined EEG/functional MRI (Fahoum et al., 2012; Pittau et al., 2012), nuclear imaging (Kim and Mountz, 2011), and cortical stimulation (Mandonnet et al., 2010; Guillory and Bujarski, 2014).

The groundbreaking concept of functional connectivity in neuroscience (Biswal et al., 1995), demonstrating that remote brain regions are functionally correlated, not only during specific tasks, but also in the resting brain, revealed that several physiological functional networks (e.g., sensorimotor, auditory, visual, attentional, salience, or default mode networks) are constantly active to a variable extent (Greicius et al., 2003), and reproducible across subjects (Smith et al., 2009). These discoveries have led to a paradigm shift in epileptology, replacing the previous concept of a purely focal seizure-onset zone with the new concept of distributed abnormal functioning in cortical and subcortical networks (Laufs et al., 2007; Gotman, 2008; Richardson, 2012; Varotto et al., 2012; Engel et al., 2013). Epileptic activity is, therefore, not an isolated local process, but occurs between connected regions (Engel et al., 2013). This paradigm shift has entered the most recent terminology of epileptic classifications by the International League Against Epilepsy (ILAE), which now considers “focal” seizures as arising “within networks limited to one hemisphere and that may be discrete or more widely distributed,” while generalized seizures are understood as “originating within or rapidly engaging, bilaterally distributed networks” (Berg et al., 2010). In focal seizures, some brain regions initiate an epileptic seizure (onset zone or “epileptogenic zone”), which is then propagated to other regions, and some regions are remotely involved, modulating or being modulated by the epileptic activity (all possibly “symptomatogenic zones”), forming, together, the epileptic network.

The interpretation of ecstatic seizures is, therefore, subject not only to the patient and his/her time and cultural background, but also to the current concepts and technical possibilities of each generation of epileptologists.

### Hypotheses on localization

In most early reports about patients presenting ecstatic auras, a temporal lobe origin was suspected, due to the emotional content and the semiology of associated symptoms (epigastric aura, complex hallucinations). Also, the Geschwind syndrome (Waxman and Geschwind, 1975), consisting of the association of hyperreligiosity, hypergraphia and hyposexuality, occurring as an interictal syndrome in some patients with temporal lobe epilepsy, could have some overlapping features with ecstatic auras (Naito and Matsui, 1988); this was hypothesized as the condition of Dostoevsky (Baumann et al., 2005). Many cases of ecstatic seizures displayed paraclinical findings suggestive of anterior temporal lobe involvement, e.g., left anterior temporal interictal discharges in the EEG (Mulder and Daly, 1952; Asheim Hansen and Brodtkorb, 2003), or an anterior temporal tumor in several cases (Mulder and Daly, 1952; Morgan, 1990; Stefan et al., 2004). Several cases had normal brain imaging; however, this information is of limited value, as the sensitivity of MRI for lesion detection has much improved in very recent years, and in the studies before the early 1990s, MRI was not available at all.

However, semiologic-anatomic correlations sometimes seemed inconsistent, e.g., in one patient, the ecstatic symptoms appeared even after the resection of the sclerotic part of the mesiotemporal region (Asheim Hansen and Brodtkorb, 2003). In another case showing hippocampal calcifications, the ecstatic semiology occurred after other ictal symptoms, suggesting a propagation to another region (Boudouresques et al., 1972). In yet another patient, the ecstatic auras appeared only after surgical resection of an epileptogenic area in the left frontal lobe (Penfield and Kristiansen, 1951), and in a fourth case, ecstatic auras disappeared after neurosurgical removal of an occipital arterio-venous malformation, although the gliotic ipsilateral hippocampus was not resected at all (Morgan, 1990). These observations seem to contradict a simple region-symptom mapping and point to a more complex semiologic-anatomic relationship, implicating a region other than the mesiotemporal area, or even several different regions within networks.

The most likely alternative region is the insular cortex. Already during Dostoevsky's ecstatic auras, laryngeal constriction was reported (Gastaut, 1984), a symptom that is nowadays recognized as quite specific for insular seizures (Isnard et al., 2004). Since the late 1940s, epileptologists have reported seizures originating from the insular cortex (Penfield and Jasper, 1954; Penfield and Faulk, 1955). However, due to the similarity of semiology between insular and mesiotemporal lobe (MTL) seizures, and because seizures of MTL origin often propagate into the insular cortex (Isnard et al., 2000), it has been nearly impossible to disentangle MTL seizures from insular seizures until recently. The multiplication of monitoring using stereo-guided insertion of depth electrodes in the insular cortex has allowed to describe the typical features of insular-onset seizures. They occur in full consciousness, can be associated with dyspnea, unpleasant somatic or perioral paresthesia, laryngeal constriction, and dysarthric speech, then can lead to a “complex” focal seizure, i.e., including impairment of consciousness (Isnard et al., 2004). In their study of electrical stimulation of implanted insular electrodes in 50 patients with temporal lobe epilepsy, Isnard et al. (2004) reported five patients out of 50 who had seizures directly originating from within the insular cortex, and one of them described symptoms of clairvoyance and mirth (case 1), suggesting an ecstatic aura, while another felt a sensation of intense warmth in the left hemibody (case 5). In other reports of patients with ecstatic auras, an increased blood flow in the insular cortex could be demonstrated, during the symptom, by the ictal SPECT (Single Photon Emission Computed Tomography) in two patients (Landtblom et al., 2011; Picard, 2013).

The presence of insular semiology does not necessarily require an insular seizure onset. It is now widely accepted that the manifestation of epilepsy is the result of epileptic activity within pre-existing neuronal wiring of a network. Not only the anatomical region of seizure onset (“onset zone” or “epileptogenic zone”) and discharge propagation, or the directly connected target areas within the network, determine the clinical presentation (“symptomatogenic zone”), but also the temporal relationship of the dynamic interplay between them during the ictal event (Chauvel and Mcgonigal, 2014). The clinical symptoms evolve with the spread of epileptic activity, not only concentrically, but also according to the specific connectivity of the onset region in micro- and macro-scale. The seizure onset zone is often not the area giving rise to the first symptoms (Rosenow and Luders, 2001), and the clinical manifestation is a complex product of activation, direct and indirect inhibition, or modulation, of often distant cortical and subcortical areas. This relationship also depends on localization: ictal discharges in the primary sensory or motor areas cause direct corresponding clinical symptoms (e.g., elementary sensory hallucinations, clonic movements) and the somato-, retino- or tono-topic organization is preserved. However when the epileptic activity occurs further up in complex, “higher” cortices, not only positive but also “negative” symptoms, i.e., extinction of the function, may occur (Chauvel and Mcgonigal, 2014).

The dense interconnection within the subparts of the insula, as well as fiber connection to the temporal, cingulate, parietal, and frontal cortex (c.f. Section Multiple Networks Allow Multi-Integrative Function of the Insular Cortex), facilitate rapid seizure propagation, from and to, insular and connected areas of the epileptic network. Depending on the exact distribution of the ictal discharge, this propagation is likely the cause for individual manifestations of ecstatic auras associated with different symptoms like olfactory, gustatory, or bodily sensations (Picard and Craig, 2009; Picard, 2013). The mesiotemporo-insular fibers serve as the main seizure propagator to the insular region (Isnard et al., 2000, 2004), which explains the often “insular” semiology of mesiotemporal lobe seizures. Following the anatomical organization, seizures in the lateral temporal neocortex can propagate to the anterior insular cortex, without going through the mesiotemporal region (Isnard et al., 2000). However, the frequent absence of the classic clinical features of lateral temporal seizures, such as visual and auditory hallucinations and illusions, or early contralateral dystonic posturing (Williamson and Engel, 2008) would argue against a primary lateral temporal origin in patients with ecstatic auras. Instantaneous spread of ictal activity between the temporal pole and the insula is suggested by recordings of synchronous spikes in these two regions (Isnard et al., 2000). No specific symptoms have been described in temporal pole seizures, except for an earlier impairment of consciousness compared to mesiotemporal seizures (Chabardes et al., 2005). Orbitofrontal seizures display complex automatisms such as violent movements and bizarre gesticulations mimicking fearful behavior, with autonomic signs, associated with an impairment of consciousness, after propagation to a larger network (Chauvel, 2003). Propagation from orbitofrontal regions also seems unlikely to explain ecstatic semiology.

Interestingly, several of the patients having ecstatic seizures report the possibility of triggering them by thinking about former fits or specific memories (pleasant and neutral; Asheim Hansen and Brodtkorb, 2003), or a pleasant emotional context, as in patient 1 from (Picard, 2013) or in other patients (personal communications, FP). This supports the idea that epilepsies have a certain reflex component with whereby a minimal level of functional activation of the epileptogenic (hyperexcitable) zone can trigger the epileptic discharge (Illingworth and Ring, 2013; Irmen et al., 2015), as especially observed in lateral temporal lobe epilepsies with auditory features, in which noises can trigger seizures (Michelucci et al., 2009), or also in the so called hot-water or bathing epilepsy (Bebek et al., 2001), in which a pleasurable ictal feeling is triggered by the strong sensory input of a hot bath, and for which abnormal insular activation has been demonstrated in hereditary forms (Nguyen et al., 2015). The case described by Cabrera-Valdivia et al. (1996) is interesting in that it links the ecstatic experience to another number of cases with idiopathic generalized epilepsy, which practice a willingly self-induction of absence seizures, e.g., by partial eye closure with upward deviation in front of a bright light, or a flickering television screen of former times (50 Hz), in order to provoke pleasurable seizures (“self-induced photosensitive epilepsy” or “television epilepsy”; Ehret and Schneider, 1961; Andermann, 1971; Binnie et al., 1980; Binnie and Wilkins, 1997). Finally, the activation of an underlying more complex specific cognitive network, which would elicit the epileptic discharge, has also been postulated for other idiopathic generalized epilepsies (Wolf, 2015).

### Ecstatic aura provoked by electrical brain stimulation

The proof of concept for the critical involvement of the anterior insula in ecstatic seizures was provided by the case of a 23 year-old woman with drug-resistant right hemispheric seizures (Picard et al., 2013b). Since the age of 12 she had reported seizures with intense feelings of bliss and well-being, like “sensations of airflow” from her stomach, with a feeling of “floating.” This was accompanied by enhanced sensory perception, especially of intense colors, and a subjective time dilation. The subsequent symptoms consisted of impairment of consciousness and gestural and oro-alimentary automatisms. During the pre-surgical evaluation, intracerebral electrodes were implanted in the right temporal lobe and insular cortex. Her seizures were found always to originate from the right mesiotemporal region and rapidly propagate to the anterior-dorsal insular cortex. However, stimulation of the right anterior-dorsal insular electrode provoked a “very pleasant funny sensation of floating and a sweet shiver” in her arms, identical to her usual auras, whereas stimulation of the right amygdala elicited strong unpleasant sensations like anxiety and epigastric pressure. None of the stimulation of other electrodes had a similar effect (Picard et al., 2013b).

In another patient, intracerebral electrodes recorded epileptic discharges in the insular cortex during a spontaneous seizure, starting with a feeling of mirth, and clairvoyance (Isnard et al., 2004). In previous studies, electrical stimulations of the insula have elicited, depending on the stimulated insular subregion, a variety of symptoms in different systems, such as gustation, olfaction, somatosensation, interoception, emotion, cognition (Penfield and Faulk, 1955; Isnard et al., 2004; Stephani et al., 2011), yet ecstatic symptoms were only reported in seven patients (Feindel and Penfield, 1954; Mullan and Penfield, 1959; Ostrowsky et al., 2000; Isnard et al., 2004; Picard et al., 2013b; Surbeck et al., 2013). A systematic collection of all studies in which intracranial electrical stimulation provoked emotional effects (i.e., 64 studies; Guillory and Bujarski, 2014) found only 12 studies reporting happiness, from which only one study actually stimulated the insular cortex (right side), inducing in one patient a pleasant sensation “as if being protected” (Smith et al., 2006). Euphoric emotions have also been provoked by stimulation of the inferior temporal gyrus, temporal pole, amygdala, inferior frontal gyrus, anterior cingulate cortex, and supplementary motor area, with a predominance in the left hemisphere (Guillory and Bujarski, 2014). However, ecstasy-like experiences are not specifically described in Guillory and Bujarski's collection. Moderately pleasant feelings have been induced by left amygdala stimulation (Lanteaume et al., 2007), while stimulation of amygdala and hippocampus have been known, for many years, to elicit very unpleasant emotions (Chapman et al., 1954; Halgren et al., 1978).

The case of our above-mentioned patient with ecstatic auras induced by electrical brain stimulation (Picard et al., 2013b) demonstrates several highly interesting facts, which advance our understanding of the mechanisms leading to ecstatic seizures in the insular cortex:

- the overwhelming feeling of an ecstatic seizure was specifically induced by the stimulation of one electrode in the right anterior-dorsal insula,
- there was no after-discharge effect due to the applied low-intensity stimulation, underlining further the very localized region for this blissful feeling,
- this region was not situated within the seizure generator zone itself, but was the symptomatogenic site of ictal propagation within the epileptic network, meaning that functional tissue alteration or degeneration is not necessarily to be expected in this region,
- this anterior-dorsal insular region likely fulfilled a similar function, before any seizure-related alteration of brain tissue occurred in this place, as suggested by the fact that the patient reported these ecstatic symptoms from the very beginning of her epilepsy.

Why were there so few cases reported in which insular stimulation provoked an ecstatic seizure, after all the years with electrical stimulation as part of appropriate presurgical evaluation in patients with epilepsy? We hypothesize that, to be generated, ecstatic auras generally need the activation of a vast region of the anterior insula, that is only rarely engaged by electrical stimulation studies, and which possibly requires a peculiar combination of ictal activation and consequent inactivation (or disinhibition) of several regions in the network of the anterior-dorsal insula (anterior-ventral insula, mid-insula, anterior cingulate cortex, orbitofrontal cortex, hippocampus, parahippocampal gyrus, amygdala, temporal pole?). The coverage of the anterior insula activated by the usual electrical stimulations could be too limited to induce such complex cognitive/emotional symptoms. In addition, although being the most direct approach to brain neurophysiology, electrical brain stimulation has some important limitations. It is difficult to estimate the extent of the brain volume that is activated by stimulation, but it is around several mm^3^. Often adjacent contacts on a given intracerebral electrode do not even record the same discharge. In addition, it is not possible to directly measure if the effect after stimulation is due to activation of the stimulated site, or to an action on a distant area or a whole sub-network remote to the stimulation site (Guillory and Bujarski, 2014). While in primary cortices clinical symptoms are very focal, specific and stereotyped, the complex association cortices, such as the insula, need a sufficient intensity of stimulation to reach a large enough extent of tissue in the vicinity, to alter a complex function. The ictal or stimulated activation directly in association cortex usually produces electrical disorganization and inhibition of function, while the ictal or stimulated activation of an associative area in a remote region might be seen as a real signal, and therefore produce the corresponding complex behavior (Chauvel and Mcgonigal, 2014).

## Neuroscientific approach of ecstatic experience

### Multiple networks allow multi-integrative function of the insular cortex

In the following section we discuss the semiology of ecstatic auras in the context of the multiple functions of the insular cortex. The insular cortex is a rather old structure, and during hominoid evolution, the anterior part has particularly undergone an important volume increase and differentiation (Nieuwenhuys, 2012; Cauda et al., 2014). The insula is cytoarchitectonically subdivided into a dorsocaudal granular zone and a rostroventral agranular zone (Kurth et al., 2009; Nieuwenhuys, 2012; Morel et al., 2013). Situated in-between the agranular and the granular zones, the dysgranular part covers the middle insula (mid-insula) and the anterior-dorsal insula (Nieuwenhuys, 2012).

The insula is widely connected, providing the anatomical bases for its integrative role, situated at the junction of allocortical functions, such as body regulation and limbic responses, and neocortical functions such as emotion and consciousness (Augustine, 1996; Craig, 2009b; Nieuwenhuys, 2012). There are abundant intra-insular connections allowing for rapid information flow (Augustine, 1996; Kurth et al., 2009). The three insular zones are connected into specific relative networks.

- The anterior-ventral insular cortex (rostroventral agranular zone) receives fibers from entorhinal cortex. It projects to the anterior cingulate cortex (ACC), temporal pole, limbic structures, medial ventral striatum, and lateral hypothalamus (Mesulam and Mufson, 1985; Augustine, 1996; Chikama et al., 1997; Ongur et al., 1998).
- The mid-insular and anterior-dorsal insular cortex (dysgranular zone) receives fibers from the primary somatosensory area and the superior temporal sulcus. It projects to the ventral prefrontal and orbitofrontal cortices, frontal operculum, pre-supplementary motor area, secondary somatosensory cortex, superior temporal sulcus, amygdaloid regions, entorhinal and perirhinal cortices, and the central and lateral ventral striatum (Mesulam and Mufson, 1982; Mufson and Mesulam, 1982; Augustine, 1996; Chikama et al., 1997).
- The posterior insular cortex (dorsocaudal granular zone) receives fibers from the primary somatosensory cortex, retroinsular area, superior temporal sulcus, basolateral amygdaloid body, entorhinal cortex and dorsolateral striatum. It projects to the supplementary motor area, ventral prefrontal cortex, secondary somatosensory area, temporal pole, retroinsular area, and dorsal thalamus (Augustine, 1996; Chikama et al., 1997).

From this connectivity pattern the peculiar situation of the insular cortex is evident, linking secondary sensory association areas with limbic areas responsible for feeding and reward (see below).

The insular cortex of humans contains so-called *von Economo* neurons (VENs), which are atypically large and spindle-shaped pyramidal neurons (Von Economo, 1926; Allman et al., 2010). They appear in elephants, cetaceans, macaques, great apes and humans, and in increasing number across the phyla (Evrard et al., 2012; Cauda et al., 2014). They are found in the anterior cingulate cortex, the anterior-ventral insula, with strong right-side predominance (Evrard et al., 2012; Cauda et al., 2014), but also in a small area of the anterior-dorsal part (Morel et al., 2013), and in the dorsolateral prefrontal cortex (Fajardo et al., 2008). The projections of the VENs in the anterior insula are known to reach ipsilateral anterior cingulate cortex and contralateral anterior insular cortex (Craig, 2009b; Allman et al., 2010); however, recently it has been suggested that the main projection site is probably more remote in the periaqueductal gray (PAG) and the parabrachial nucleus (PBN) (Evrard et al., 2012), two regions that receive interoceptive afferents from the spinothalamic tract, and coordinate the specific autonomic patterns of cardiovascular, respiratory and motor responses to relevant stimuli as well as pain transmission (Benarroch, 2012; Butti et al., 2013). This identification of a projection of the anterior-ventral part of the insula to the PAG (and hypothalamus) is crucial regarding its visceromotor autonomic function (see below).

**Figure 2:**
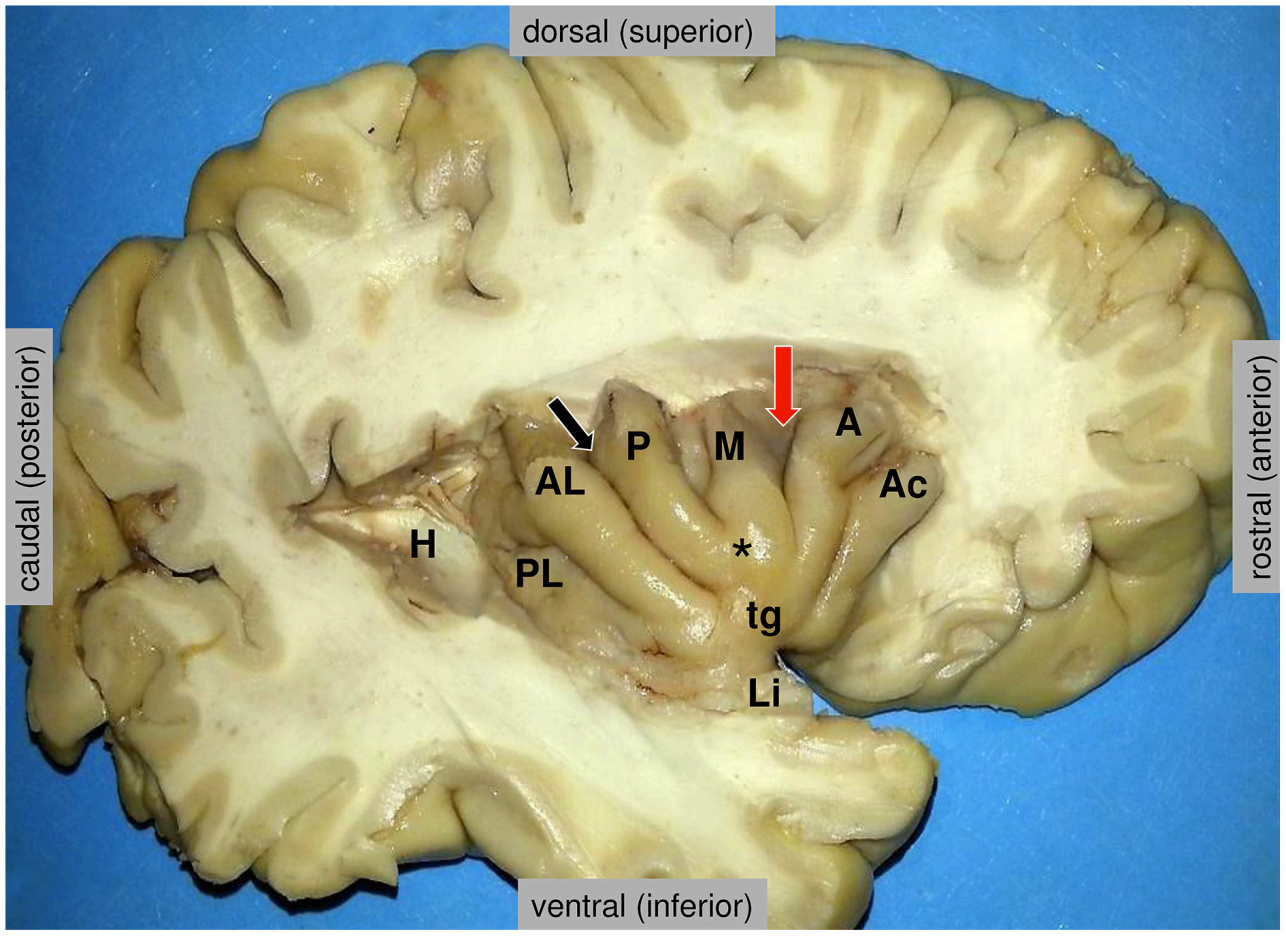
**Anatomy of the insula, as disclosed in the depth of the lateral fissure**. H = the posterior medial stub of the transverse temporal gyrus of Heschl (primary auditory cortex) that was resected to uncover the posterior long insular gyrus. The central sulcus (black arrow) divides the lateral surface of the insula into a small posterior insular lobule, composed of the anterior long (AL) and posterior long (PL) insular gyri that converge to the limen insulae (Li), and a large anterior insular lobule, composed of the anterior short (A), the middle short (M) and the posterior short (P) insular gyri that converge to the apex of the insula (^*^). The anterior face of the insula displays a variably present accessory insular gyrus (Ac) and a constant transverse insular gyrus (tg) that connects with the orbital surface of the frontal lobe. The red arrow marks the sulcus between the anterior short and middle short gyri, where the functional “overlap region” was found by Kurth et al. (2010). Figure courtesy of Drs. Thomas P. Naidich and Mary E. Fowkes, the Icahn School of Medicine at Mt. Sinai, New York.

Coinciding with its wide connections in many different networks, the insula is implicated in a large number of different brain functions (Augustine, 1996; Craig, 2009b; Nieuwenhuys, 2012). According to a functional meta-analysis, they can be grouped into four functional domains (Kurth et al., 2010), defined as (1) a social-emotional domain (anterior-ventral insula) for emotion and empathy, (2) a cognitive domain for attention, speech production and language, (3) an olfacto-gustatory domain and (4) a sensorimotor domain including interoception, somatosensation, pain and motion. All these categories, except somatosensation and motion, show a remarkable overlap on the anterior-dorsal insula, precisely located at the dorsal end of the sulcus between the middle and anterior short gyri (Kurth et al., 2010; Figure 2, red arrow). Another meta-analysis identified the same main subdivisions, with posterior, ventro-anterior and dorso-anterior regions, corresponding to sensorimotor, affective/chemosensory, and cognitive processing, respectively (Chang et al., 2013). In a large sample of 355 participants, the persistent concordance between structural, functional, and connectivity-based parcellation of both (left and right) insular cortices was demonstrated (Kelly et al., 2012).

The overlap in the mapping of the categories in the anterior-dorsal insula could be due to a basic functional role that all categories have in common, which suggests a high hierarchical role of this region (Kurth et al., 2010). There is a gradient in connectivity from simpler connected regions in the posterior insular regions to the highly complex connected regions in the anterior-dorsal insula (Cerliani et al., 2012), and the anterior-dorsal insula was therefore regarded as the final stage of a hierarchical processing, starting in the posterior insula with pure sensory information, then integrating emotional and cognitive valuation, ending in the anterior-dorsal insular region with a full representation of a “sentient self,” the sine qua non of self-awareness (Craig, 2009b, 2010; Kurth et al., 2010). The VENs are well suited for rapid long-distance integration of information (Allman et al., 2010) and are regarded as critically involved in autonomic regulation (Butti et al., 2013). They may play a major role in interoception, i.e., the perception of the physiological body states (Craig, 2002), and emotional awareness at the pivotal point between bodily states and conscious behavior (Gu et al., 2013). Besides, it has been noted that the VENs are found in species with a highly developed social life (Allman et al., 2010; Critchley and Seth, 2012; Cauda et al., 2014).

### Bliss, clarity and intense serenity—emotion regulation, and prediction error

Ecstatic seizures are above all extraordinary emotional experiences, which in some cases can be “life-changing.” Among the ictal experiences of emotion, the large majority are unpleasant and of elementary nature, such as anxiety and fear (60%), and depression (20%), while ictal joy is only rarely reported (Johanson et al., 2008).

**Figure 3:**
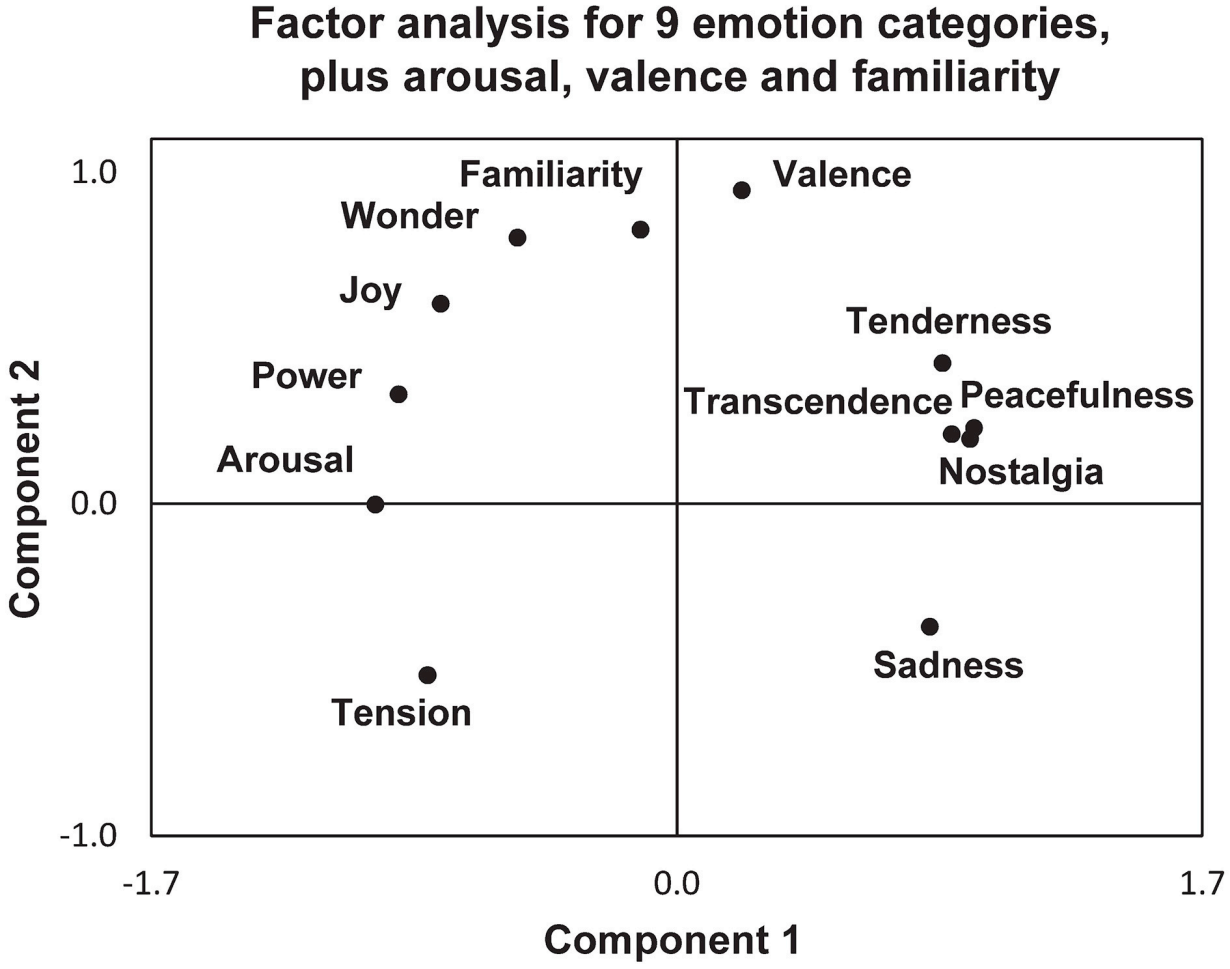
**A factorial analysis of emotional ratings after listening to different pieces of music, including the nine emotional categories of the Geneva Emotional Music Scale (GEMS) model (Zentner et al., 2008), together with familiarity, arousal and valence, reprinted with permission from Trost et al. (2012)**. The results show two main components, arousal (***component 1***) and valence (***component 2***), that best describe these behavioral data in 31 participants.

Different models of emotions have been proposed: the classic emotion model with a small number of basic emotions such as happiness, sadness, disgust, fear, anger, and surprise (Ekman and Cordaro, 2011), or the conceptualization of emotions along the dimensions of arousal and valence (Russell and Barrett, 1999). In their approach to map more emotions elicited while listening to different types of music, Trost et al. (2012) provided an integrative diagram of complex feelings like tenderness, peacefulness, transcendence, nostalgia, familiarity, wonder or power along the two dimensions of valence and arousal (Figure 3).

Following the concept of two orthogonal components arousal and valence, in-between which complex emotions can be localized, we suggest that the emotional semiology of an ecstatic seizure be situated (a) at the utmost positive valence: all patients underline the extraordinary blissful moment. As to the degree of arousal during ecstatic auras, however, there is a certain paradoxical situation: many patients report simultaneously both high arousal emotions like power or joy, and low arousal emotions like peacefulness or transcendence. Despite the peacefulness, patients describe that they feel extraordinarily alert and highly aware of the “here and now” during the episodes. We suggest therefore that (b) the semiology of ecstatic seizures is not obviously correlated with the degree of arousal, according to the categories proposed in this model. The reason for this ambivalence in arousal appraisal can possibly be found in the unusual emotional nature of the ecstatic state itself, “never felt in everyday life.”

Different neuroimaging studies have shown that insula and amygdala responses predominantly reflect stimulus valence interacting with intensity and arousal effects (Winston et al., 2005). There is a so-called “negative bias,” meaning that aversive stimuli normally are experienced with higher intensity and more arousal, whereas positive stimuli rather induce varying levels of arousal (Krolak-Salmon et al., 2004). While the insula has mostly been associated with the experience or recognition of negative emotions, particularly the disgust (Jabbi et al., 2008), complex emotions of the highest positive valence, like maternal or romantic love, have also been shown to activate the anterior insular cortex (AIC) and anterior cingulate cortex (ACC) (Bartels and Zeki, 2004; Leibenluft et al., 2004), or pleasant and mesmerizing musical moments activated the insula and regions of the dopamine reward system such as striatum and ventral tegmental area (Salimpoor et al., 2011). In a meta-analysis across 162 neuroimaging studies of emotion and affect, Kober et al. (2008) identified six groups of constantly coactivated regions (networks), which could be linked to distinct emotional components (including attention to emotional signals, and visual processing). Among these groups, the “core limbic” and the “lateral paralimbic” groups contain the most attributes of the arousal and valence components of affective experience. The “core limbic” group included amygdala/left hippocampus, thalamus, PAG and hypothalamus. The “lateral paralimbic” group comprised the anterior-dorsal insula, anterior-ventral insula/orbitofrontal cortex (OFC), posterior-ventral insula, ventral striatum, and temporal pole. The “medial prefrontal cortex” (“medial PFC”) group, encompassing ACC, was directly connected to the “lateral paralimbic” group, and both groups (but only the ventral insular regions of the “lateral paralimbic” group) were closely associated with the “core limbic” group.

The awareness of emotion is strongly linked to interoception, the sensation of bodily states. The relationship between the physiological body states and the emotional state was already described by James and Lange in the early 20th century. The James-Lange theory of emotion pointed out that emotions result from physiological body reactions to external events, which was later described as the “somatic marker hypothesis” (Damasio, 1996; Craig, 2004). The insula is a major cortical location processing the autonomic signals (Craig, 2002, 2009b), with a viscerosensory (afferent) component in the posterior insula (for interoception processing) and a visceromotor (efferent) component in the anterior-ventral insula. The insula is therefore one of the favored structures where emotional processing takes place: along the posterior-to-anterior re-representation within the insula, the processing integrates the interoception in the posterior insula progressively with cognitive and motivational information in the anterior insula.

Based on ideas of theoretical neurobiology, the awareness of emotions has recently been conceptualized as “interoceptive inference,” where the subjective feelings are based on the cognitive evaluations and predictions of changes of the body states (Seth et al., 2011; Seth, 2013). A perpetual process of predictions is generated and extrapolated from the present state and is then continually compared to the actual incoming signals, generating prediction errors and an update of the next predictions. The aim is always to resolve uncertainty and minimize prediction errors (Picard and Friston, 2014). Based on a predictive processing on interoception, the anterior insular cortex has been suggested to play the role of the comparator of the predicted to the actual outcome in this evaluation loop in the context of feeling states (Singer et al., 2009; Seth et al., 2011; Seth and Critchley, 2013) and the anterior insula was shown to be particularly activated in anticipation of aversive events, in different studies (Ploghaus et al., 1999; Nitschke et al., 2006). During risky decisions, e.g., in gambling tasks, the anterior insula encodes the risk prediction while waiting for the outcome. Once the outcome is known, it generates the risk prediction error, by acting as a comparator between predicted risk and realized risk (Preuschoff et al., 2008; Singer et al., 2009), allowing the possibility for the learning of risk prediction. In the reinforcement learning theory, the learning of negative value of loss-predicting signals seems to involve the anterior insula (“avoidance learning”) while the learning of positive value (“approach learning”) occurs in the ventral striatum (Palminteri et al., 2012).

In some neuropsychiatric disorders, for example in patients with anxiety disorders (Feinstein et al., 2006; Paulus and Stein, 2006), the processes of decision-making in uncertainty have been shown to be altered, toward an intolerance to uncertainty and ambiguous situations, which generates avoidance behavior. Patients appear to have altered interoceptive prediction signals, with abnormally increased anticipation of aversive stimuli, correlating with enhanced anterior insula activity (Paulus and Stein, 2006). Also in obsessive-compulsive disorder, which is characterized by a high subjective experience of doubt (in contrast with the sense of certainty experienced by the patients during ecstatic auras), patients showed a greater activation of the anterior insula during anticipation of aversive stimuli in a fMRI study (Jung et al., 2011), while they have a larger gray matter volume in the anterior insular cortex (Nishida et al., 2011; Song et al., 2011), and changes in connectivity in anterior insula and dorsal anterior cingulate cortex (Cocchi et al., 2012).

In summary, the insula plays a role in negative and positive emotion experience, but most of all in anticipation of emotions, with a large body of literature on its role in anticipation of aversive events (Preuschoff et al., 2008; Liu et al., 2011; Skvortsova et al., 2014) and generation of anticipatory somatic markers of such events (Yu et al., 2010). It will then initiate adaptive responses to prediction error by adapting the autonomic reflexes for a relevant behavior, and allow optimal updates of the next interoceptive predictions, in order to resolve uncertainty.

During ecstatic seizures, some patients report a sense of clarity and clairvoyance, and some explain that “everything seems to be exactly in the right place, as it should be.” One patient (patient 1, Picard, 2013) even explained that, while listening to a discussion between several people during an ecstati aura, he felt as though he had a sudden full understanding of the topic, “as an access to the solution.” We hypothesize that in the ecstatic auras the mechanisms of interoceptive prediction error generation are blocked, the comparator between the actual and the predicted state no longer functions, and that there is no more mismatch. This may lead for a few seconds to a pathologically-induced (epileptic) “stable state” without any generated prediction error, without ambiguity, causing a feeling of certainty and intense serenity and inner peace (Picard, 2013; Picard and Kurth, 2014).

### Heightened self–awareness and environment perceptions—salience and experience of time

Ecstatic seizures are dominated by a feeling of a conscious self-presence, characterized by a heightened self-awareness, and a feeling of union with the world, the “All,” with breakdown of the barrier between the subject and surroundings. The anterior insula participates in the self-reflective network, maintaining a coherent first-person perspective, on the basis of its connections toward inferior parietal lobe and temporo-parietal junction (Augustine, 1996; Craig, 2009b; Modinos et al., 2009; Dennis et al., 2014; Ionta et al., 2014). Somatosensory stimuli, as other internal and external stimuli, are processed in the posterior insula and cognitively integrated more anteriorly in the insula (Craig, 2003). An ictal hyperactivation of this region will alter this processing and may provoke abnormal unpleasant feelings (Isnard et al., 2004), or in rare cases very pleasant ones (Williams, 1956; Stefan et al., 2004), with sometimes even a feeling of sensory overload, as some patients report.

**Figure 4:**
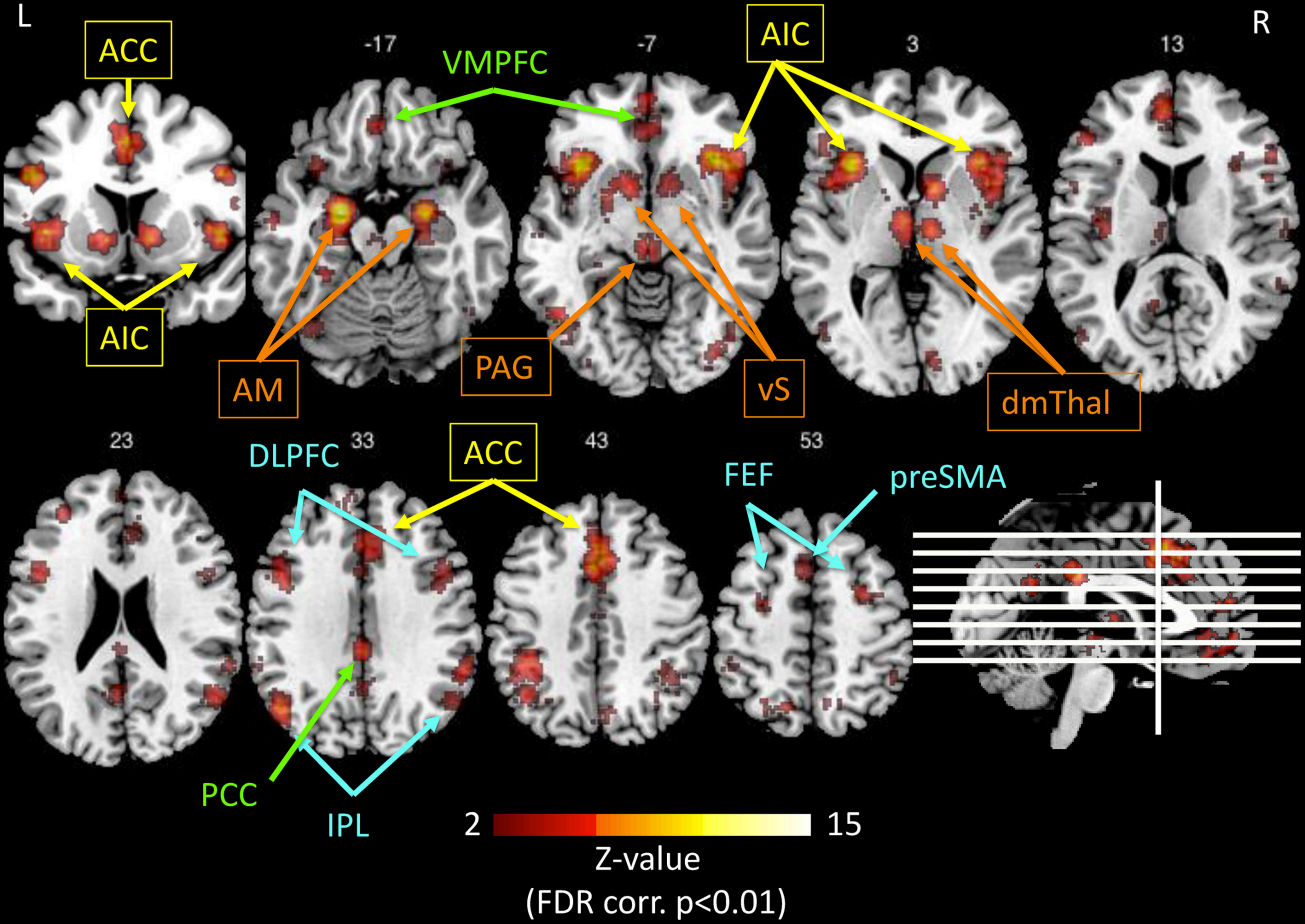
**Illustration of the networks implicated in salience processing, as obtained by the meta-analysis tool Neurosynth.org (Yarkoni et al., 2011), based on the results of 122 published studies, using the only keyword “salience” in forward inference, and displayed on a template anatomical T1 image with Z-values of 2 to 15 (using the Mango software package^2^)**. The resulting significant regions (*p* < 0.01, FDR corrected) interestingly comprise four networks: Yellow: anterior cingulate cortex (ACC) and anterior insula (AI) represent the salience network (Seeley et al., 2007b). Orange: the extended insular network consisting of amygdala (AM), ventral striatum (vS), periaqueductal gray (PAG), and dorsomedial thalamus (dmTHal). Green: the PCC-VMPFC represents the default mode network (Greicius et al., 2003). Blue: the fronto-parietal executive control network (Corbetta and Shulman, 2002; Fox et al., 2006). The salience network is thought to switch back and forth between the default mode network and executive control network (Menon and Uddin, 2010; Uddin, 2015).

Probably in connection with its role in interoception and autonomic function, the anterior insula is specifically sensitive to salient internal and environmental events, i.e., behaviorally-relevant stimuli, together with the dorsal anterior cingulate cortex (dACC) in the “salience network” (Seeley et al., 2007b; Eckert et al., 2009; Menon and Uddin, 2010; Wiech et al., 2010; Uddin, 2015). This network detects and evaluates salient signals in order to continuously resolve uncertainty and adapt the behavior, by driving autonomic reflexes (which in turn engage feelings). As already mentioned in Bliss, Clarity, and Intense Serenity—Emotion Regulation and Prediction Error, the final aim is to minimize prediction errors and maintain homeostasis. The role of the salience network comprises the switching between different mind states, particularly between the default mode network (Greicius et al., 2003) and the executive control network (Figure 4; Sridharan et al., 2008; Hasenkamp et al., 2012; Tang et al., 2012; Chang et al., 2013). Conjoint activation of the anterior insula and the ACC was also recently demonstrated during effortful cognitive tasks (Engstrom et al., 2014).

It seems relevant that an ictal hyperactivity in the salience network may add a feeling of importance to any stimulus (internal or external), giving rise to a feeling of heightened perception of the (body) self and of the neighborhood (e.g., colors of the environment). This paradox of the heightened self-awareness in combination with a feeling of union with the world, could be related to an intensification of the “sentient self” and a functional extinction of the “narrative,” autobiographical self, which is prominent during mind wandering and related to the activity of the default mode network (Hasenkamp et al., 2012). This “isolation” of the sentient self would allow to intensely experience the present moment, with intensified interoceptive and exteroceptive perceptions (Craig, 2009b; Picard and Craig, 2009; Picard, 2013; Picard and Kurth, 2014).

When every moment is labeled “salient,” the perceived time flow appears stretched. The temporal judgment of the “nowness,” and the estimation of an ongoing duration, have been shown to be tightly linked to the function of interoception and to emotional processing (Craig, 2009a,b; Wittmann, 2011), and is modulated by the sentient processing, arousal, and attention (Schirmer, 2011). Several imaging studies have confirmed insular implication in time judgments (Coull, 2004; Livesey et al., 2007; Stevens et al., 2007; Van Wassenhove et al., 2008; Wittmann et al., 2010; Wittmann, 2013). Craig proposed that at each moment, there is a posterior to anterior insular integration of interoceptive, sensory and emotional information into a “global emotional moment” (Craig, 2009a,b). The succession of these moments produces a cinemascopic “image” of the sentient self, which serves as a basis for time perception, each “global emotional moment” lasting around 125 ms (Picard and Craig, 2009; Wittmann, 2013). However, the sampling rate of this integration is modulated by the salience of the input. The salient moments increase the sampling rate and lead to a subjective dilation of time (Craig, 2009a). In an ecstatic aura, when each moment is perceived as salient, the overwhelmingly high number of consecutive salient moments would increase the sampling rate to a maximum, leaving the patient subjectively timeless in a sustained state of “present-moment awareness” (Craig, 2009b; Picard and Kurth, 2014).

Very similar feelings as those in ecstatic auras are reported after consumption of stimulant drugs such as e.g., cocaine, amphetamine, or ecstasy (Picard and Kurth, 2014). Moreover, a state of enhanced awareness of the present moment with minimized mind wandering is one of the general aims of most meditation techniques. It involves a cognitive reappraisal of emotionally salient sensory events. Functional brain imaging studies have reported that activation in the anterior insula during meditation correlated with the self-reported intensity of meditation and was higher in advanced meditators (>10'000 practice hours) compared to beginners (Lutz et al., 2008; Tang et al., 2012), and that modulation of *state anxiety* by mindfulness meditation engaged a network of brain regions including the anterior insula (Zeidan et al., 2014). In the advanced stage of training, meditation is maintained by activity in the left insula, the anterior cingulate cortex, and the striatum (Tang et al., 2012). Structural imaging studies have shown a higher gray matter concentration (Holzel et al., 2008), in a thicker cortex (Lazar et al., 2005), with stronger gyrification, in the anterior insula in experienced meditators compared to controls (Luders et al., 2012).

### Comparison to insular loss-of-function pathology and right/left considerations

Given the ictal neuronal hyperactivation during a seizure, it is also useful to look at the semiology of conditions causing neuronal deficit in the same regions. A loss-of-function pathology for example by stroke or a tumoral lesion in the posterior and middle insula can cause loss of bodily feelings like analgesia or thermoanesthesia (Craig, 2002, 2009b), anosognosia for hemiplegia or asomatognosia (Vocat et al., 2010), and even hemispatial neglect (Manes et al., 1999; Karnath et al., 2004; Rousseaux et al., 2013). A selective depletion of the VENs in the anterior insula and anterior cingulate cortex has been demonstrated in patients with fronto-temporal dementia, who present a lack of self-conscious emotions (Seeley et al., 2006, 2007a; Allman et al., 2010; Evrard et al., 2012). There is a constantly growing awareness that the insular cortex is implicated in many psychiatric syndromes (Goodkind et al., 2015), such as borderline personality (Nagai et al., 2007), anxiety and mood disorders (Feinstein et al., 2006; Piguet et al., 2014; Wiebking et al., 2015), schizophrenia and autism spectrum disorders (Kasai et al., 2003; Allman et al., 2005).

Ecstatic seizures have been reported with brain lesions or electroencephalographic findings in both hemispheres. So far, there is no clear argument in favor or against lateralization in either direction (right, *n* = 21; left, *n* = 16; undefined, *n* = 12; see Table 1). Whereas lesion studies of patients with altered bodily schemata, such as anosognosia for hemiplegia (Vocat et al., 2010), or hemispatial neglect (Karnath et al., 2004; Rousseaux et al., 2013) naturally concentrate on the right hemisphere and therefore right insula, the left/right asymmetry in emotional disturbances of epileptic origin is less clear. Among the many focal epilepsy cases collected by Williams (Williams, 1956), several reported simultaneous ictal feelings of joy and depression, or pleasure and fear in individual mixtures during the same aura, and patient 2 in (Picard, 2013) also suffered from unpleasant emotional seizures (sense of dread) before the ecstatic seizures, reflecting the modularity of these experiences but also the probable topographical neighborhood (or connections, e.g., insular left/right) for these various emotional feelings. Bi-directional functional connectivity between left and right anterior insulae could be identified after stimulation, with a very short latency of 10–20 ms for passing from one to the other (Lacuey et al., 2015).

A hemispheric specialization was proposed for emotional valence, toward a left-sided positive valence and a right-sided negative valence (for review, see Craig, 2009a,b, 2011). One recent meta-analysis of 143 studies (*n* = 2721 participants) using emotional tasks (Duerden et al., 2013) gave results that are consistent with these findings: while negative stimuli activated the whole insula bilaterally, positive stimuli showed a left-hemisphere dominance in the anterior and mid-insula. It is noteworthy that both above-mentioned studies found significant differences between male and female participants: males processed emotional stimuli more in the left anterior and the right posterior insula, whereas females activated bilateral anterior insula and left posterior insula (Duerden et al., 2013). Actually left anterior insula seems to be activated by all valence categories (pleasant/positive and unpleasant/negative), while the right anterior insula would only encode negative feelings (Gu et al., 2013). In Guillory and Bujarski's meta-analysis of a collection of 64 electrical stimulation studies (Guillory and Bujarski, 2014), the relationship between elicited positive and negative emotional responses was also clearly in favor of euphoric-left (ratio of 9:1 studies) and dysphoric-right (ratio of 8:1 studies) lateralization.

The reason for the valence lateralization probably lies in the insular organization: it has been hypothesized that the laterality of brain activity associated with emotions could originate in differences in autonomic input to and output from the left and right insular cortices, as postulated by Craig (2002), with a predominance of parasympathetic activity on the left side and of sympathetic activity on the right side (Oppenheimer et al., 1992; Oppenheimer, 2006; Craig, 2009a,b, 2011). These differences could be integrated in a concept of opponent emotional control systems: the regulation of energy nourishment in the left forebrain, and the operationalization of energy expenditure in the right forebrain (Craig, 2005; Evrard and Craig, 2015).

## Further directions

Although, there seems to be convincing evidence, explaining ecstatic seizures as emerging from an epileptic network depending mainly on the anterior-dorsal insular cortex, there are still many open questions. During our discussions with patients we noted some peculiarities of the ecstatic state: a sensation of total inner peacefulness, together with, at the same time, the highest arousal level; a feeling of unity with the universe at the same time with the highest feeling of self-awareness. Already the early works on emotional seizures have revealed the possible very narrow coexistence of pleasurable and unpleasurable emotions. Depending on the patients, there can be symptoms that are more self-centered (inner peace, heightened bodily perception) or more oriented toward the external world (hyperperception of the external world, union with the universe). Could this, as well as differences in valence, possibly be related to differences in predominating activity in the left or the right insula? Or could there be differences according to the insular subregions involved, as shown for the anterior cingulate cortex where a rostral subregion was activated by reward and a caudal subregion was activated by negative aspects of reward processing (Liu et al., 2011)? Do the VENs play a specific role in emotion generation and in ecstatic phenomena? What are the major neurochemical systems involved in emotion processing? The role of the most famous candidate for pleasure, the dopamine, involved in predicting reward (Schultz et al., 1997), is unclear in generating the sensation of pleasure *per se* (Berridge and Kringelbach, 2015). The involvement of the opioid and the orexin systems has been recently reported in appetitive reactions (Mahler et al., 2014; Berridge and Kringelbach, 2015), but the neurochemical processes involved in emotion experience are far from being elucidated. As the number of nicotinic receptors is especially high in the anterior-dorsal insular cortex (Picard et al., 2013a), we wonder about the role of the nicotinic system in autonomic and emotion processing in the anticipation and experience of aversive stimuli, and possibly in the generation of pleasure.

To better test our hypothesis of transitory interoceptive and emotional prediction error suppression during ecstatic auras, the combination of fMRI (or SEEG with insular electrodes) and experimental paradigms using aversive learning (e.g., Seymour et al., 2004; Palminteri et al., 2012) could be used in either the patients with ecstatic auras, or in meditators or individuals under stimulant substance consumption experiencing ecstatic phenomena. Another track could be to compare insular activity in such paradigms in patients with anxiety disorder (Paulus and Stein, 2006), compared to self-assured participants at peace, e.g., meditators.

Last, but not least, improving the understanding of the fascinating ecstatic seizures would unarguably help to untie some of the most complex mechanisms of emotion processing in self-awareness and value generation. The paradoxical fact that an abnormal neuronal activity with dysregulated synchronization induces the highest and most peaceful mental state was very precisely reflected by Dostoevsky's character Mishkin, in The Idiot:

[…] he had often said to himself that all those flashes and glimpses of a higher self-sense and self-awareness, and therefore of the “highest being,” were nothing but an illness, a violation of the normal state, and if so, then this was not the highest being at all but, on the contrary, should be counted as the very lowest.

*And yet he finally arrived at an extremely paradoxical conclusion: “So what if it is an illness?” he finally decided. “Who cares that it's an abnormal strain, if the result itself, if the moment of the sensation, remembered and examined in a healthy state, turns out to be the highest degree of harmony, beauty, gives a hitherto unheard-of and unknown feeling of fullness, measure, reconciliation, and an ecstatic, prayerful merging with the highest synthesis of life?” These vague expressions seemed quite comprehensible to him, though still too weak (Dostoevsky*, *1869**, p. 226).*

This remarkable paradox explains the general fascination for the ecstatic auras. Similar experiences might have influenced religious beliefs over time, and therewith have had an impact on the history of human culture.

## Funding

MG was supported by the Swiss National Science Foundation No. 33CM30-124115. FP by the Swiss National Foundation No. 320030-127608.

### Conflict of interest statement

The authors declare that the research was conducted in the absence of any commercial or financial relationships that could be construed as a potential conflict of interest. The Associate Editor VM declares that, despite of being affiliated with the same institution as the author MG, the review process was handled objectively.

## Footnotes

^1^Photograph publicly available under https://commons.wikimedia.org/wiki/File:Dostoevsky.jpg

^2^Available online at: http://rii.uthscsa.edu/mango/index.html
